# Society Proceedings

**Published:** 1890-03

**Authors:** 


### SOCIETY PROCEEDINGS.

*The Ohio State Veterinary Medical Association* held its seventh annual meeting in the Neils Post Hall, Columbus, O., Jan. 15th, 1890.

The President, Dr. T. B. Hillock, called the meeting to order at 2 P.M.

The attendance was small. What the meeting lacked in numbers, however, was amply compensated for by the vigor and enthusiasm of those present.

The following officers were elected for the ensuing year :—

President, Dr. G. W. Butler, Circleville; First Vice-President, Dr. T. B. Colton, Mt. Vernon; Second Vice-President, Dr. J. D. Fair, Berlin; Third Vice-President, Dr. W. R. Howe, Dayton; Secretary, Dr. W. J. Torrance, Cleveland; Treasurer, Dr. B. Hillock, Columbus.

The Board of Censors were suspended until a written motion for the abolition of the said Board might be voted upon at the next meeting of the association.

Dr. L. S. Snyder, Coshocton, O., was unanimously elected to membership.

The Secretary presented the official correspondence, which contained a set of resolutions prepared by Dr. T. Butler, of Davenport, Iowa, requesting our Association to urge the United States Veterinary Medical Association to meet in future in some Western city, with first preference Chicago; second preference, St. Louis.

These resolutions were tabled, and it was decided that we should urge the United States Association to be our guests at Dayton. O. Dr. Howe (Ohio Secretary of United States Veterinary Medical Association), was requested to use his influence in furthering the desired object.

Dr. J. S. Butler, of Piqua, O., read an able paper on "Roaring," discussing the subject in all its phases and describing the operation for the cure of the same, as he had lately performed it upon three cases. One of the cases had proven a positive success. The other two were apparently equally successful, but the subjects had not yet been put to their final tests. Dr. Butler's paper received well-merited applause from those present.

The annual report of the Treasurer was audited and showed a thriving condition of the finances of the Association.

A discussion was now opened upon Dr. Butler's paper and upon the details of his operations by Drs. Shaw, Wight, Howe, George W. Butler, J. D. Fair, T. B. Colton and others.

Dr. Torrance reported an unsuccessful operation which he performed upon a roarer.

Spasmodic roaring, spasm of the glottis, choking, etc., etc., were now discussed and cases relating thereto were reported by Drs. Gribble, G. W. Butler, T. B. Colton and J. D. Fair.

Reports of cases were continued for the remainder of the afternoon session, some of the more interesting of which were as follows: Dr. Torrance reported a case of rupture of the diaphragm; also a case of a dead fœtus being retained by a mare for over a year in that condition. Dr. J. S. Butler reported a similar case to the latter. Dr. Gribble reported the removal of a mummified six-months' old bull calf which had been retained two years in utero. Dr. Colton reported a similar case due to torsion of uterus, in which the mare still carries the foal. Dr. Gribble referred to the prevalence of tetanus among the suckling foals of his county, which Dr. George Butler claimed was due to non-cicatrization of the umbilicus. Paralysis of the pharynx and similar operations were discussed by Drs. Gribble, Fair, J. S. Butler and Torrance. Dr. George Butler reported the removal of a fifteen pound tumor from the vagina of a cow and referred to the effects of tracheotomy in the prevention of persistent straining in the cow. The humanity of the custom of dehorning cattle, and similar subjects were discussed by Drs. Gribble and T. B. Colton.

Dr. Yonkerman's charges of "breach of etiquette" against Dr. Shaw were tabled and Dr. Shaw was completely exonerated.

The following committees were appointed by the President:—

Committe on Contagious Diseases, Drs. Colton, J. C. Myer and J. S. Butler; Committee on Veterinary Progress, Drs. J. D. Fair, T. B. Hillock and Gribble.

The Secretary was instructed to correspond with Dr. Salmon with the hope of procuring a copy of the last report of the U. S. Bureau of Animal Industry for each member of our Association.

Reports of cases were again discussed. Dr. J. S. Butler spoke of pelvic abscesses, and reported a case of paralysis due to melanosis of the post-aorta. Dr. Torrance spoke of necrosis following electric shocks and of melanosis producing lameness. Dr. G. W. Butler reported a case of eversion of vagina in a yearling filly, probably due to a shock of lightning. Dr. Colton described a peculiar disease prevalent upon the river bottoms of Nebraska. Dr. Gribble reported a chronic case of eversion of vagina in a mare. Dr. Wight reported a case of intussusception in a horse where two feet of the ileum were found in the cæcum. Drs. Wight and Torrance reported cases of rupture of the rectum. Drs. J. S. Butler and T. B. Hillock reported cases of rupture of uterus in mares.

A few more cases were reported and the meeting adjourned.

Next meeting to be held at Dayton, Ohio.

WILFRED J. TORRANCE,
*Secretary.*

---

*Indiana Association of Veterinary Graduates.*—The annual meeting of the Indiana Association of Veterinary Graduates was held in the Agricultural Room at the State House, Indianapolis, on the evening of September 25th, 1889, the President, Dr. H. R. Macaulay, in the chair.

After the usual preliminary business had been despatched, the election of officers for the ensuing year took place. Dr. M. E. Knowles, of Terre Haute, was elected president; First Vice-President, Dr. J. M. Curphey, of Noblesville; Second Vice-President, Dr. C. F. Bell, of Kokoma; Third Vice-President, Dr. W. B. Wallace, of Marion; Secretary, Dr. H. R. Macaulay, of Indianapolis; Treasurer, Dr. J. C. Rodgers, of Anderson; Board of five Trustees, Drs. Bolser, Galbraith, Ferling, Upshall and Ward.

Following the election came the reports of committees, and then Dr. Knowles addressed the meeting on the "Causes and Treatment of Sterility." The subject was very ably handled and made very comprehensive by the display of instruments used.

Dr. Curphey then read an interesting paper on "Canine Distemper," which called forth a lively discussion, after which the meeting adjourned for the evening.

The continued session was held on the morning of the 26th, with Dr. Knowles, President, in the chair. During this part of the meeting there were animated and instructive discussions on cases presented by the following members on the following subjects: Dr. Diggs reported a case of tetanus and one of paralysis; Dr. Thompson, one of tetanus; Dr. Shoemaker, locomotor ataxia; Dr. Macaulay, phrenitis; Dr. Mylne, poll evil; Dr. Rodgers, a peculiar case of eczema.

The meeting then adjourned.

H. R. MACAULAY, *Secretary.*

---

*New York State Veterinary Medical Society.*—Meeting for organization held in the Chamber of Commerce, Rochester, N. Y., Jan. 15th, 1889. When the meeting was first called it was intended to be a meeting of the veterinary surgeons of Western New York, but when veterinary surgeons flocked into the committee room from points as far West as Buffalo and as far East as Amsterdam, it was an easy matter to denominate the meeting a State affair, and the organization effected before adjournment was accordingly called: "The New York State Veterinary Medical Society."

The object of the meeting was to perfect an organization having for its principal mission the securing of legislation that will protect qualified practitioners in the practice of their profession and prevent unqualified persons setting up as veterinary surgeons. When the meeting was called to order the following veterinary surgeons were present: W. G. Dodds, Canandaigua; H. E. Rowell, Albion; J. G. Hill, Sennett; Joseph Suterby, Le Roy; H. C. Klecher, Canadea; B. Howes, Brockport; Harry Suterby, Batavia; W. P. Hinkley, Buffalo; E. Bowen, Seneca Falls; James Carnite, Amsterdam; D. K. Seltzer, Penn Yan; A. L. Hunter, Watkins; John A. Bell, Watertown; W. G. Hollingworth, Utica; Wilson Huff, Rome; John Wenda, Buffalo; M. M. Poucher, Oswego; O. B. French, Honeoye Falls; C. C. Willard, Mount Morris; Dr. Whyte and Dr. Albert Drinkwater, of Rochester; Dr. Whytock, Warsaw; Dr. McQueen, Hornellsville; Dr. Stevenson, Tyre; Dr. G. H. Roberts, Akron.

Dr. Suterby, of Batavia, called the meeting to order, and Dr. Drinkwater, of this city, nominated Dr. Claude D. Morris, of Bath, for chairman, and he was unanimously chosen.

Four irregular practitioners who had drifted into the meeting under the impression that they belonged to the profession were requested to leave. They objected to leaving, asserting that they had registered their names in the office of the county clerk at an expense of $2, and they thought they had just as much right to doctor a horse as a man who had a diploma from a veterinary college. But the meeting did not think so, and the quartette took the elevator to the street, and the meeting went on harmoniously. The following named officers were elected: President, Dr. Claude D. Morris, of Bath; Vice-President, Dr. Albert Drinkwater, of Rochester: Secretary, W. P. Hinkley, of Buffalo; Treasurer, W. G. Dodds, of Canandaigua. An adjournment was then taken until 1.30 P. M.

At the afternoon session censors and committees were appointed as follows:—

Censors--Drs. Wenda, of Buffalo; Suterby, of Batavia; Whytock, of Warsaw; Hunter, of Watkins, and Bell, of Watertown.

Committees—Arrangements for next meeting, Drs. McQueen, of Hornellsville; Poucher, of Oswego; Hinkley, of Buffalo. Publication, Drs. Hinkley, Dodds, Rowell, Huff and Stevenson. Legislation, Drs. Morris, Hinkley, Hollingworth. By-laws, Drs. Hinkley, French, Whyte. Consultation, Drs. Roberts, Joseph Suterby, Frank Suterby, Seltzer, Carnite.

The Society decided to meet semi-annually, and Syracuse was selected as the permanent place for holding the meeting. The initiation fee was fixed at $5 and the annual dues at $2. The important committee is the one on legislation, and it has been instructed to draft a bill for presentation to the Legislature, setting forth the qualifications necessary to entitle a man to practice as a veterinary surgeon and imposing severe penalties upon unqualified persons attempting to practice. President Morris made the startling statement that there are 750 unqualified persons in this State posing as veterinary surgeons. Of this number 350 were unable to write their names and were obliged to make an "x" when registering at the county clerk's office.

A recommendation that the Society become incorporated and secure a charter, made by President Morris, was adopted.

A brief session was held in the evening, at which President Morris read a proposed act to raise the standing of the veterinary corps of the Unity States Army. The act was ordered spread upon the minutes and the Society will use its influence to secure its favorable consideration by Congress.

On motion of Dr. Hollingworth, tuberculosis was placed on the same contagious list with pleuro-pneumonia.

A banquet was immediately held in the lunch room of the Chamber of Commerce, which was attended by nearly all who had attended the business session. It was a very pleasant affair. When the wine was reached, President Morris made a graceful little speech, thanking the press of Rochester for its courtesies and concluded by asking those present to drink to the health and prosperity of the Rochester press. "Let us do it as we used to in College" said President Morris, and immediately there was a clinking of glasses and shouts of "Press! Press! Press!" The banquet was brought to a close at half past 10 o'clock.

---

*The Keystone Veterinary Medical Association* held its monthly meeting at the College of Physicians, Philadelphia, February 1st, 1890. Dr. Huidekoper presided. The following members were present: Drs. Hoskins, Huidekoper, Weber, Rayner, Kooker, Williams, Zuill, Lintz, Cullen, Eves, Glass, Harger, Ridge, L. Lusson, Magil and Bachman. There were also several visitors present.

The minutes of the January meeting were read. Dr. Zuill asked to have the resolution offered by him at the last meeting stricken from the minutes, which was granted; otherwise the minutes were approved. There not being a quorum of the Board of Trustees present, the President appointed Drs. Lusson, Rayner and Ridge to fill the vacancies. Dr. Birch's application for membership was considered and reported favorably to the Association. The Legislative Committee reported arrests in Union County for violation of registry law, also legal proceedings in other counties. Dr. Hoskins reported the false arrest of Dr. Kooker (one of the Committee on Legislation) for slander—based upon statements made by another person in a suit against an irregular practitioner. It was moved that the Keystone Association bear Dr. Kooker's expenses of defense, which was carried. The committee on place of meeting reported that they had rented the room of the College of Physicians,[1] of Philadelphia, N. E. corner of 13th and Locust Streets. This report was accepted, and a vote of thanks extended to the committee.

Dr. Zuill moved that in the discussion of papers and cases that the members shall confine themselves to the subject under discussion, and that no member shall speak more than ten minutes, nor shall he speak twice on the same subject without permission from the chair.

Dr. Birch was elected by ballot. Drs. Eves and Cullen were appointed tellers; they reported thirteen votes cast, all being in favor of his election.

Dr. Huidekoper read the bill for the organization of an Army Veterinary Corps formulated by the Military Committee of the U. S. Veterinary Medical Association. It was approved by the Keystone Veterinary Medical Association. The essayist, Dr. Keelor, being absent, the report of cases was responded to by Dr. L. Lusson, who reported four cases—parturient apoplexy, calcification of cervical ligaments, a case of broken back, and a case of azoturia—which brought out a lively discussion. Dr. Lusson stated that he cured his case of parturient apoplexy by giving all the whisky the owner had, and then gave turpentine, and thought the stimulant treatment was the proper treatment. Dr. Ridge reported a case where he gave tincture aconite all through the disease, even when the case was comatose and cold, but the cow recovered. Dr. Bachman reported a bad case where he gave nothing but water and his case got well.

[1] This room is used by the County Medical Society, Pathological, Obstetrical and almost all of the medical societies of Philadelphia.

There was a diversity of opinion in regard to hot pack to the loins, in azoturia. The question arose whether we always had coffee-colored urine in azoturia. Dr. Glass said he had seen some cases where the urine was only slightly colored, but yet was not normal. Dr. Hoskins said the coffee-colored urine was pathognomonic, and unless he got it he would be very slow to call it azoturia. Dr. Williams favored Dr. Glass, while Dr. Kooker thought we could have no azoturia without coffee-colored urine.

Dr. Huidekoper reported two cases—first, a case of odontomes taken from a horse, one on each side of upper jaw, half way between molars and tusk. The horse had wolf-teeth. The specimens were passed around. Dr. Hoskins reported a similar case. The next case was a commuted fracture of the hyoid bone. The case resembled strangle, and the autopsy revealed the lesion.

There were several visitors present. Dr. Rose, of the Department of Agriculture, made a few remarks. He said that contagious pleuro-pneumonia did not exist in this State; that he had examined several reported cases, but found them to be tuberculosis, which existed to some extent.

W. H. RIDGE, *Secretary.*

---

*Long Island Veterinary Society.*—A regular meeting of the *Long Island Veterinary Society* was held on February 19th, at 74 Adams Street, Brooklyn, New York., the President, Dr. George H. Berns in the chair. The following members were present: Drs. George H. Berns, R. R. Bell, R. A. McLean, George F. Bowers, E. Hanshew, Jr., Samuel Atchison, H. H. Housman, D. S. Breslin, and J. F. Musloe. The minutes of the previous meeting were read and approved.

The Board of Censors made no report. The next order of business was the reading and discussion of papers. As the discussion of the paper read at the previous meeting of the society had been postponed until this meeting, Dr. R. A. McLean re-read his paper on influenza, which elicited an animated and instructive discussion from all the members present, particularly Drs. R. R. Bell, George H. Berns, R. A. McLean, George F. Bowers and J. F. Musloe.

Dr. R. R. Bell reported to the society on behalf of the Dean of the American Veterinary College a request that the society select one of its members to act as one of a board of three examiners to examine applicants for the practical prize of the College. The request was received and accepted by the society and Dr. George F. Bowers was unanimously selected to act as the representative of the society on said board.

Dr. R. A. McLean proposed the name of Dr. James McKeen, of No. 14-16 Nevins Street, for membership of the society. Meeting then adjourned.

D. S. BRESLIN, D.V.S., *Secretary.*

